# A role for the yeast CLIP170 ortholog, the plus-end-tracking protein Bik1, and the Rho1 GTPase in Snc1 trafficking

**DOI:** 10.1242/jcs.190330

**Published:** 2016-09-01

**Authors:** Cécile Boscheron, Fabrice Caudron, Sophie Loeillet, Charlotte Peloso, Marine Mugnier, Laetitia Kurzawa, Alain Nicolas, Eric Denarier, Laurence Aubry, Annie Andrieux

**Affiliations:** 1 Univ. Grenoble Alpes, Grenoble F-38000, France; 2 Inserm, U1216, Grenoble F-38000, France; 3 CEA, BIG, Grenoble F-38000, France; 4 Institute of Biochemistry, Department of Biology, ETH Zurich, Zurich 8093, Switzerland; 5 Institut Curie, Recombinaison et Instabilité Génétique, CNRS UMR3244, Université Pierre et Marie Curie, Paris Cedex 75048, France; 6 Inserm, U1038, Grenoble F-38000, France

**Keywords:** Microtubule, +Tips, Tyrosination, Trafficking, CLIP170, Rho1

## Abstract

The diversity of microtubule functions is dependent on the status of tubulin C-termini. To address the physiological role of the C-terminal aromatic residue of α-tubulin, a *tub1-Glu* yeast strain expressing an α-tubulin devoid of its C-terminal amino acid was used to perform a genome-wide-lethality screen. The identified synthetic lethal genes suggested links with endocytosis and related processes. In the *tub1-Glu* strain, the routing of the v-SNARE Snc1 was strongly impaired, with a loss of its polarized distribution in the bud, and Abp1, an actin patch or endocytic marker, developed comet-tail structures. Snc1 trafficking required dynamic microtubules but not dynein and kinesin motors. Interestingly, deletion of the microtubule plus-end-tracking protein Bik1 (a CLIP170 ortholog), which is preferentially recruited to the C-terminal residue of α-tubulin, similarly resulted in Snc1 trafficking defects. Finally, constitutively active Rho1 rescued both Bik1 localization at the microtubule plus-ends in *tub1-Glu* strain and a correct Snc1 trafficking in a Bik1-dependent manner*.* Our results provide the first evidence for a role of microtubule plus-ends in membrane cargo trafficking in yeast, through Rho1- and Bik1-dependent mechanisms, and highlight the importance of the C-terminal α-tubulin amino acid in this process.

## Abstract (precis)

**Summary:** In the yeast *Saccharomyces cerevisiae*, cargo trafficking involves microtubule plus-ends and a Bik1-dependent mechanism that requires the presence of the C-terminal aromatic residue of β-tubulin.

## INTRODUCTION

Microtubules are fibrous structures in eukaryotic cells that play a vital role in cell organization and division. From yeast to human, the C-terminal residue of α-tubulin is a highly conserved aromatic residue (tyrosine in most mammalian cells; phenylalanine in *S. cerevisiae*). In mammals, microtubules are subjected to detyrosination and tyrosination cycles, during which the C-terminal aromatic residue of α-tubulin is removed from the peptide chain by an as yet unidentified carboxypeptidase and then re-added to the chain by a tubulin tyrosine ligase (TTL). This process generates two pools of tubulin: tyrosinated α-tubulin and detyrosinated α-tubulin with an exposed glutamate at the tubulin end (known as detyrosinated-tubulin or Glu-tubulin). Tubulin tyrosination has many important functions. For example, TTL loss, which results in the accumulation of Glu-tubulin, confers a selective advantage to cancer cells during tumor growth (Kato et al., 2004; Mialhe et al., 2001), and TTL suppression in mice leads to a lethal disorganization of the neuronal circuits (Erck et al., 2005). In a previous work, we generated a budding yeast strain solely expressing an α-tubulin devoid of its C-terminal aromatic residues (*tub1-Glu* strain) to model detyrosinated Glu-tubulin, as re-addition of phenylalanine is not observed in the *tub1-Glu* mutant cells (Badin-Larcon et al., 2004). Using this strain, we discovered that the CLIP170 ortholog Bik1 is able to sense the C-terminal α-tubulin aromatic residue at microtubules plus-ends (Badin-Larcon et al., 2004). This feature is conserved in mammalian cells for all the plus-end tracking CAP-Gly-domain-containing proteins, including CLIP170 (also known as CLIP1) (Peris et al., 2006). Structural studies have established that the C-terminal aromatic residue is required for the direct interaction of α-tubulin with CAP-Gly domains and CLIP170 (Honnappa et al., 2006; Mishima et al., 2007).

To further investigate the physiological role of microtubule tyrosination, we performed a synthetic-lethality-based screen to identify genetic partners of Glu-tubulin in budding yeast. This approach revealed that *tub1*-*Glu* mutant cells have a strong and specific requirement for a small set of genes associated with vesicular trafficking and related processes. Study of the v-SNARE Snc1 trafficking in the *tub1-Glu* mutant revealed a marked misrouting defect of the protein. We demonstrated that Bik1 is involved in Snc1 trafficking. We further showed that a constitutively active form of Rho1 promotes the loading of Bik1 onto microtubule plus-ends and restores a proper Snc1 trafficking in the *tub1-Glu* strain.

Overall, this work shows the power of the synthetic lethality screen approach in revealing, in the yeast model *Saccharomyces cerevisiae*, unexpected functions of microtubule plus-ends, and more specifically of the C-terminal residue of α-tubulin.

## RESULTS

### A genome-wide screen for Glu-tubulin specific lethality

To identify new functions of the α-tubulin C-terminal amino acid, we challenged the viability of the *tub1-Glu* mutation in a collection of strains individually deleted for the 4847 non-essential genes using a 96-well microplate format and a robotic liquid-handling system (Loeillet et al., 2005). Around 50 genes essential for the normal growth of *tub1-Glu* strain were identified and seven were confirmed for synthetic lethality or growth defect using manual dissection (Table S1). Namely the histone variant H2AZ *HTZ1*, the transcriptional repressor *TUP1*, the mannosyltransferase *MNN9*, the endosomal protein *CDC50*, the protein kinase *VPS15*, the geranyl-geranyl diphosphate synthase *BTS1* and the 1-3-β-D-glucan synthase *FKS1* were found to be required for the normal growth of the *tub1-Glu* strain. To derive hypotheses regarding biological functions required for the survival of *tub1-Glu* cells, the genetic partners were grouped according to their biological functions. Surprisingly, none of these genes were revealed to be microtubule components or known partners, but five of the seven genes were found to belong to gene ontology categories referring to intracellular protein transport, endocytosis and the Golgi. To date, the role of microtubules in endocytosis and related trafficking aspects in yeast has been poorly documented (Huffaker et al., 1988; Jacobs et al., 1988; Kubler and Riezman, 1993; Penalver et al., 1997). These results derived from the synthetic lethality screen prompted us to re-investigate this question in more details with a special focus on the C-terminal amino acid of α-tubulin.

### The C-terminal residue of α-tubulin is crucial for Snc1 trafficking and for proper Abp1 localization

**Fig. 1.**
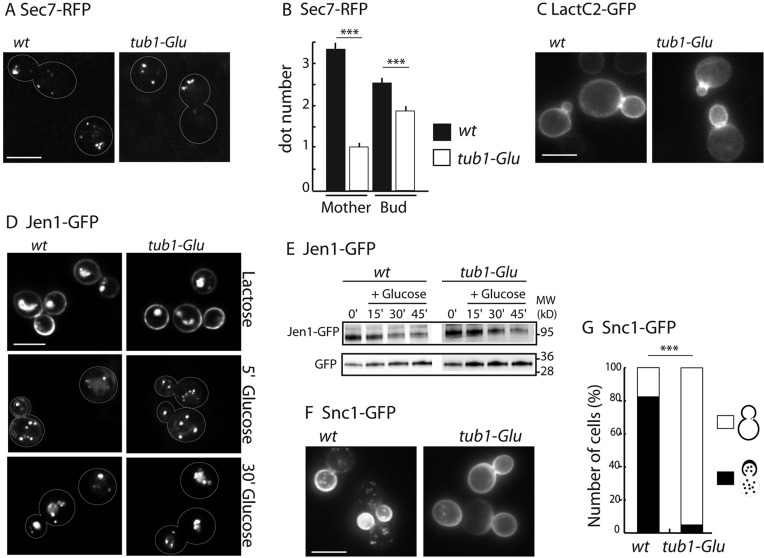
**Glu-microtubules impair Snc1 routing.** (A,B) Analysis of the trans-Golgi in Sec7–RFP-expressing cells. (A) Sec7–RFP localization in *wt* and *tub1-Glu* strains. (B) Quantification of Sec7–RFP dot number [mean±s.e.m., *n*=51 cells and for wild-type (*wt*) and 42 cells for *tub1-Glu*]. ***P<0.0001 (two-tailed Mann and Whitney test). (C) Localization of phosphatidylserine detected using the GFP–LactC2 probe. In both strains, the fluorescence signal of GFP–LactC2 was largely confined to the plasma membrane and concentrated at incipient bud sites. (D,E) Analysis of the glucose-induced endocytosis of the Jen1 transporter. Parental and *tub1-Glu* cells harboring Jen1–GFP at endogenous chromosomal locus were grown in lactate medium. Jen1–GFP subcellular localization (D) and degradation (E) were monitored following glucose addition by fluorescence microscopy or by immunoblotting using anti-GFP antibody, respectively. No significant differences were observed between strains. (F,G) Localization of GFP-tagged Snc1 expressed from a low copy plasmid. (F) Snc1 localization in *wt* and *tub1-Glu* strains. (G) Quantification of the Snc1–GFP signal in *wt* and *tub1-Glu*. The protein was observed either at the bud plasma membrane and in cytoplasmic dots, or at mother and bud plasma membranes. The percentage of cells in each category is shown (*n*=90 cells for *wt* and 175 cells for *tub1-Glu*). ***P<0.0001 (Fisher's exact test). Scale bars: 5 µm.

Previous data based on the use of thermosensitive mutants of tubulin or microtubule-destabilizing drugs has shown that there is a role for the budding yeast microtubular network in Golgi organization. We first questioned the possible requirement of the C-terminal aromatic residue of microtubules in this function by analyzing the distribution of the ARF guanine nucleotide exchange factor Sec7, a marker of the trans-Golgi, in the *tub1-Glu* strain. Analysis of trans-Golgi Sec7–RFP-positive punctae revealed that the average number of Sec7–RFP-positive vesicles was significantly reduced in the *tub1-Glu* mutant compared to the wild-type (*wt*) strain, most particularly in the *tub1-Glu* mother cells (Fig. 1A,B). This result corroborates the previously published defect in trans-Golgi organization induced by microtubule destabilization (Rambourg et al., 1996). Additionally, as the *tub1-Glu* mutation is not responsible for major defects in terms of microtubule length and dynamics (Caudron et al., 2008), our data are strongly indicative of a specific role for the C-terminal residue of α-tubulin in trans-Golgi organization.

We next investigated whether vesicular trafficking requires an intact α-tubulin. To this aim, we analyzed the impact of the *tub1-Glu* mutation on the behavior of three GFP-tagged constructs used herein as reporters to follow the integrity of the endocytic and secretory pathways: the phosphatidylserine-binding C2 domain of the lactadherin protein (LactC2), the yeast lactate transporter Jen1 and the v-SNARE Snc1.

In yeast, phosphatidylserine is synthesized in the endoplasmic reticulum and delivered to the plasma membrane by trans-Golgi derived secretory vesicles. This anionic lipid, as followed by use of the phosphatidylserine-specific GFP–LactC2 probe, first concentrates at the site of bud formation, as a consequence of polarized membrane trafficking towards the daughter cell (polarized exocytosis), and then accumulates at the bud neck and the bud itself (Fairn et al., 2011). In both *wt* and *tub1-Glu* strains, GFP–LactC2 was enriched at the bud cortex and at the bud neck (Fig. 1C), indicating that polarized exocytosis is not notably affected in the *tub1-Glu* strain, despite a possible disorganization of the Golgi network. Accordingly, growth and budding, which require active membrane delivery, are grossly normal in the *tub1-Glu* strain (Badin-Larcon et al., 2004), as they are after microtubule destabilization using cold-sensitive β-tubulin or nocodazole (Huffaker et al., 1988; Jacobs et al., 1988).

The lactate transporter Jen1 becomes highly enriched at the plasma membrane when lactate is used as the sole carbon source in the medium. Upon addition of glucose, the permease is internalized by endocytosis and targeted to the vacuole for degradation after transiting through the trans-Golgi network (Becuwe et al., 2012). This degradation can be followed by live imaging of cells expressing Jen1–GFP with the loss of the protein at the plasma membrane and the progressive accumulation of fluorescence in the lumen of the vacuole (Fig. 1D). In western blot analysis, this leads to the disappearance of the fusion protein from the whole-cell extract and the accumulation of GFP, a degradation product of Jen1–GFP resistant to the vacuolar hydrolysis activity (Fig. 1E). In the *tub1-Glu* mutant, glucose addition led to the degradation of the protein with kinetics similar to that observed in *wt* cells, indicating that the mutation has no major effect on Jen1 trafficking and the plasma-membrane–endosome–Golgi–vacuole route. These results correlate with data from two other groups showing that disruption of the microtubule network using β-tubulin mutants or nocodazole treatment had no effect on the endocytosis of the yeast maltose transporter and α-factor receptors in response to signals similarly triggering their targeting to and degradation in the vacuole (Kubler and Riezman, 1993; Penalver et al., 1997).

Snc1 functions on trans-Golgi derived secretory vesicles as a key player controlling their fusion with the plasma membrane. GFP–Snc1 accumulates at the cell surface, from where it recycles back to the trans-Golgi by endocytosis after sorting at the endosome level (Lewis et al., 2000). During budding, Snc1 localizes preferentially at the bud plasma membrane, due to polarized exocytosis and active endocytosis that prevents its diffusion to the mother cell membrane (Valdez-Taubas and Pelham, 2003). Accordingly, in *wt* budding cells, GFP–Snc1 was found to localize essentially to cytosolic vesicles (endosomes or trans-Golgi) and to the bud plasma membrane (Fig. 1F,G). In contrast, in *tub1-Glu* cells, the percentage of cells with a polarized GFP–Snc1 localization was reduced (5% in the *tub1-Glu* versus 82% in the *wt*; Fig. 1F,G). In a large proportion of the *tub1-Glu* cells, GFP–Snc1 distributed at the plasma membrane of both the bud and the mother cell, with a reduced number of GFP–Snc1 vesicles in the cytoplasm, suggesting that the C-terminal aromatic amino-acid of α-tubulin is needed for proper trafficking of Snc1 along the plasma-membrane–endosome–Golgi– plasma-membrane route.

**Fig. 2.**
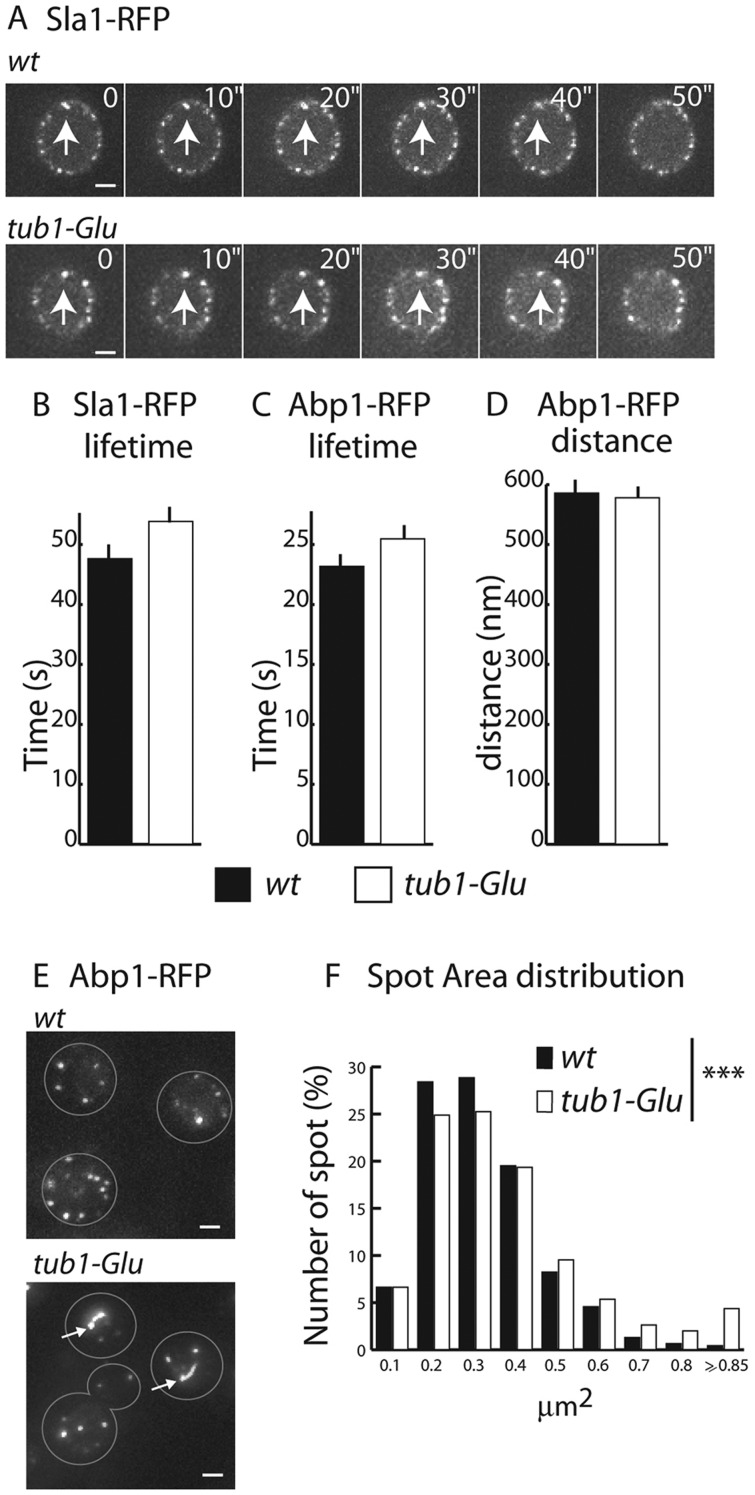
**Abp1 accumulates in comet tail structures in the *tub1-Glu* strain.** (A) Dynamic behavior of Sla1–RFP in *wt* and *tub1-Glu* strains. The arrow highlights patches that appeared and disappeared over time. Sla1p–RFP (B) and Abp1–RFP (C) patch lifetime and Abp1–RFP patch traveled distance (D) are shown. No significant differences are observed (for *wt* and *tub1-Glu*, respectively: Sla1, *n*=311 and 345 tracks; Abp1, *n*=415 and 508 tracks). (E) Representative images of Abp1–RFP localization in *wt* and *tub1-Glu* cells. Arrows indicate comet tail structures. (F) Quantification of the surface area of Abp1–RFP-positive spots for *wt* and *tub1-Glu* strains. For each strain, the percentage of spots in each category is shown (*n*=461 and 1085 spots for *wt* and *tub1-Glu*, respectively). Abscises values are the center of each class of size. Distributions are significantly different. ***P<0.0001 (χ^2^ test). Scale bars: 1 µm.

As a loss of Snc1 polarized distribution was frequently observed in mutants harboring defects in the endocytic machinery (Burston et al., 2009), we wondered whether the *tub1-glu* mutation could limit or affect the internalization step, thereby impairing the recycling efficiency of Snc1. Snc1 internalization has been shown to involve a clathrin- and actin-dependent pathway (Burston et al., 2009). As actin is the key player in membrane invagination and clathrin-coated vesicle formation, forming endocytic vesicles are visible as cortical actin-positive patches upon phalloidin staining. This qualitative analysis indicated that actin patches were similar in size and distribution in the *wt* and tub1-Glu strains (data not shown). We then followed, by live imaging, the dynamics of two relevant indicators of the membrane invagination and vesicle budding steps, the proteins Sla1 and Abp1. Sla1 is recruited very early during clathrin coat maturation at the endocytic sites, whereas Abp1 appears later as the actin meshwork organizes around the forming vesicle. The two proteins are removed rapidly after vesicle budding. As reported previously (Kaksonen et al., 2005), Sla1 and Abp1 fused to RFP were found to localize to discrete cortical puncta that continuously formed and disassembled in both *wt* and *tub1-Glu* cells (Fig. 2A). The dynamics of these Sla1- and Abp1-positive puncta, as quantified by automated analysis using the Icy software, was not significantly affected by the *tub-Glu* mutation compared to the *wt* (Fig. 2B–D). However, and very strikingly, besides the discrete cortical patches, the *tub1-Glu* strain often displayed aberrant Abp1 staining on larger patches or comet tail structures as shown in Fig. 2E (arrows) and quantification of the surface area of all Abp1-positive dots, patches and comets clearly indicates a bimodal distribution in the *tub1-Glu* strain compared to the *wt*, with the presence of a population of larger structures (Fig. 2F, size ≥0.7 µm^2^). Abnormal staining patterns of Abp1 in comet tails was previously observed in mutants lacking players in the clathrin-mediated endocytosis machinery (Kaksonen et al., 2003; Newpher et al., 2006; Prosser et al., 2011) and could indicate a partly impaired internalization in the *tub1-Glu* mutants. Such defects could noticeably impact upon Snc1 distribution and explain the Snc1 mislocalization in the *tub1-Glu* mutant, as Snc1 enrichment at the bud requires an efficient and persistent recycling process to maintain its polarized distribution.

### Snc1p trafficking requires dynamic microtubules

**Fig. 3.**
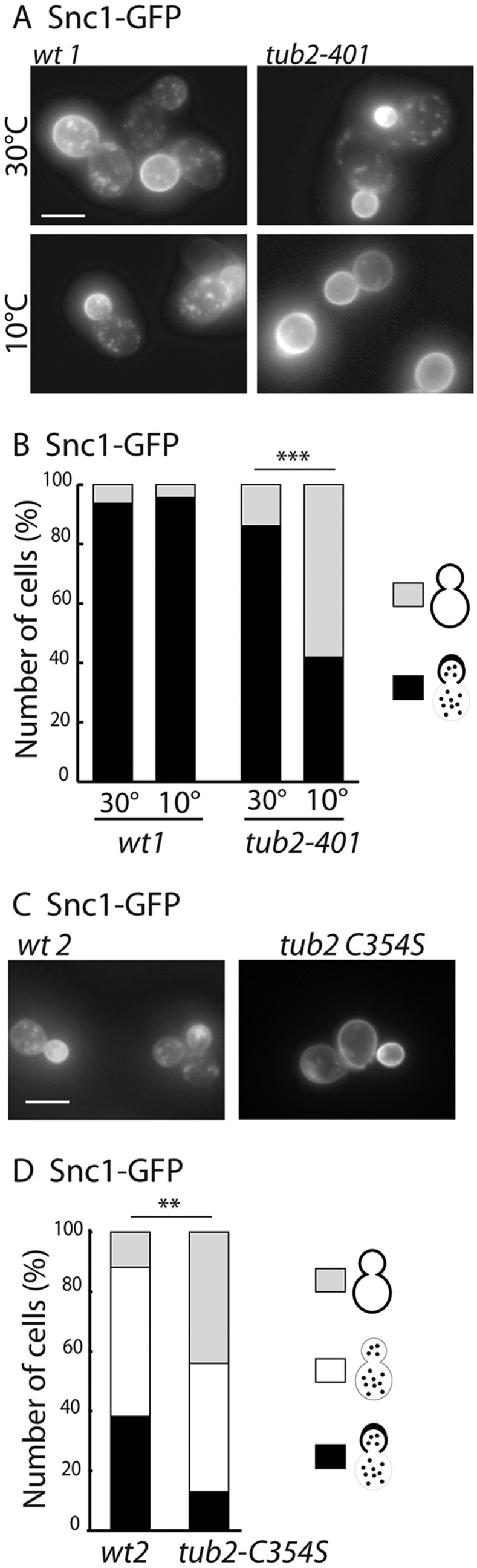
**Snc1 sorting defects are observed in cold-sensitive or non-dynamic microtubules.** (A,C) Localization of GFP-tagged Snc1 expressed from a low copy plasmid. (A) Snc1 localization in *tub2-401*, a microtubule cold-sensitive strain and isogenic control strain (*wt1*). The cells were exponentially grown at 30°C and shifted to 10°C for 1 h. (B) Quantification of the Snc1–GFP signal in *wt1* and *tub2-401* strains. The percentage of cells in each category is shown (for 30°C and 10°C, respectively: *wt1*, *n*=32 and 35 cells; *tub2-401*, *n*=46 and 62 cells). The protein was detected either at the bud plasma membrane and in cytoplasmic dots, or at mother and bud plasma membranes. ***P<0.0001 (Fisher's exact test). (C) Snc1 localization in the *tub2-C354S* strain, which has a decreased microtubule dynamicity, and isogenic control strain (*wt2*). (D) Quantification of the Snc1–GFP signal in *tub2-C354S*. Compared to B, an additional staining was observed corresponding to a uniform distribution on cytoplasmic dots. The percentage of cells in each category is indicated (*wt2*, *n*=34 cells; *tub2-C354S*, *n*=38 cells). **P<0.001 (χ^2^ statistic test). Scale bars: 5 µm.

In the *tub1-Glu* strain, as both microtubules and free tubulin dimers are modified, we tried to define which defect (Glu-tubulin or Glu-microtubules) was interfering with Snc1 trafficking. To that aim, we tested the involvement of microtubules using a cold-sensitive *tub2-401* mutation of the sole gene encoding β-tubulin in *S. cerevisiae.* At the restrictive temperature, this mutation induces the destabilization of the microtubule network and results in the absence of assembled microtubules (our data not shown, and Huffaker et al., 1988). Whereas *tub2-401* mutant cells kept at permissive temperature harbored a distribution of GFP–Snc1 similar to that observed in *wt* cells, shifting the cells to 10°C for 1 h, led to the loss of the polarized localization of GFP–Snc1 with a noticeable enrichment at the mother cell cortex (Fig. 3A,B). Such treatment had no effect on the *wt* strain, indicating that the microtubule network is required for efficient transport of Snc1.

Microtubules are highly dynamic structures, and we wondered whether such dynamics were required for Snc1 trafficking. To address this question, we used a *tub2-C354S* mutant strain that strikingly dampens microtubule dynamicity *in vivo* and *in vitro* (Gupta et al., 2002). In the *wt* genetic background corresponding to this strain, GFP–Snc1 distribution was different from that of other *wt* strains as only 40% of the cells displayed an enrichment of GFP–Snc1 at the bud (Fig. 3C,D). In the *tub2-C354S* strain, the polarized GFP–Snc1 population was reduced to 13%, as compared to 40% in the *wt* strain*.* Concomitantly, the population of cells harboring staining at the mother and bud plasma membranes reached 44% in the *tub2-C354S* strain versus 12% in the *wt*, supporting a requirement for microtubule dynamics in Snc1 trafficking (Fig. 3C,D). These results indicate that the role of the C-terminal aromatic residue of α-tubulin in proper trafficking of the v-SNARE protein Snc1 is therefore likely to take place in the context of dynamic microtubules.

### Snc1 trafficking involves the plus-end-tracking protein Bik1

**Fig. 4.**
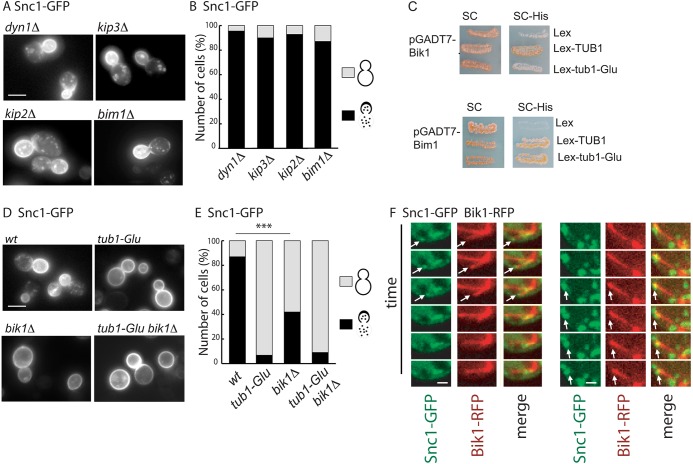
**Bik1 is required for Snc1 traffic.** (A) Localization of GFP–Snc1 in *dyn1Δ*, *kip2Δ*, *kip3Δ* and *bim1Δ* strains. (B) Quantification of the localization pattern either at the bud plasma membrane and in cytoplasmic dots, or at mother and bud plasma membranes (*dyn1Δ, n*=44; *kip2Δ*, *n*=46; *kip3Δ*, *n*=42; *bim1Δ*, *n*=50 cells). (C) Two-hybrid interaction between the +Tips Bik1 and Bim1 (fused to the GAL4 activation domain in pGADT7 plasmid) tested against *wt* tubulin (*TUB1*) and tubulin lacking the final C-terminal residue (*tub1-Glu)* fused to the LexA DNA-binding domain in the pLex plasmid. The colonies were striated onto SC plates lacking uracil and leucine (SC) or SC plates lacking histidine (SC-His) to detect interaction after 3 days of growth at 30°C. Bik1 interacts with *TUB1* and not with *tub1-Glu*, whereas Bim1 interacts with both tubulins. (D) Localization of GFP-tagged Snc1 in *wt*, *tub1-Glu*, *bik1Δ* and *tub1-Glu bik1Δ*. (E) Quantification of the localization pattern of GFP-tagged Snc1 in the different strains. The percentage of cells in each category is shown (*wt*, *n*=62; *tub1-Glu*, *n*=50; *bik1Δ*, *n*=127 cells; *tub1-Glu bik1Δ*, *n*=45 cells). ***P<0.0001 (Fisher's exact test). (F) Montage of six images acquired sequentially of *wt* cells expressing Bik1–RFP to label microtubules and GFP–Snc1 to label vesicles, taken over a 6-s period. Images correspond to a stack of relevant *z*-images. Arrows indicates vesicle and microtubule coordinated movement. Scale bars: 5 µm (A,D), 1 µm (F).

In mammals, microtubules contribute to endocytic vesicle motility through the molecular motors of the dynein and kinesin families. We thus questioned whether such motor proteins were involved in microtubule-driven Snc1 trafficking. The distribution of GFP–Snc1p was analyzed in the *dyn1*Δ *s*train devoid of the sole gene encoding the heavy chain of the dynein motor in *S. cerevisiae*. Snc1 localized similarly to *wt* in the *dyn1*Δ mutant cells (Fig. 4A,B). Similar results were obtained with mutants devoid of the kinesins *KIP2* or *KIP3*, known to function antagonistically in the microtubule-dependent positioning and movement of the nucleus (Cottingham and Hoyt, 1997). Our observations therefore indicate that the role for microtubules in Snc1 trafficking is not crucially dependent on the Kip2 and Kip3 kinesin and Dyn1 dynein molecular motors.

As the α-tubulin C-terminal amino acid has been shown to be crucial for the interaction of CLIP170 and the yeast ortholog Bik1 with microtubule plus-ends through their CAP-Gly domain (Badin-Larcon et al., 2004; Honnappa et al., 2006; Peris et al., 2006), we investigated a possible role for Bik1 in mediating the effect of the *tub1-Glu* mutation. As expected from published data (Schwartz et al., 1997), Bik1 interacted with the *wt* α-tubulin in two-hybrid experiments whereas interaction with Glu-tubulin was barely detectable (Fig. 4C, upper panels). We next analyzed the localization of GFP–Snc1 in a mutant strain deleted for *BIK1*. In the *bik1Δ* strain, the distribution of GFP–Snc1 was reminiscent of that observed in the *tub1-Glu* strain with a loss of polarity and an increase in the localization of the protein at the mother cell plasma membrane (Fig. 4D,E). The disruption in the *tub1-Glu* strain with deleted *BIK1* did not worsen or alter the defects in GFP–Snc1 trafficking, an observation in favor of a role for these two proteins in the same genetic pathway. In contrast to Bik1, deletion of the yeast EB1 ortholog Bim1, another member of the plus-end-tracking protein family (known as +Tips) but which does not have a CAP-Gly domain and whose interaction with α-tubulin is independent of the C-terminal aromatic residue (Fig. 4C, lower panels) did not impede Snc1 trafficking (Fig. 4A,B). Our data therefore demonstrate a crucial and specific role for the +Tip CAP-Gly domain in Bik1 for Snc1 trafficking.

Given the enrichment of Bik1 at the plus-end of microtubules, we next investigated whether Snc1-positive vesicles were able to move in a coordinated manner with microtubule plus-ends. Live-cell imaging was performed on *wt* cells expressing Snc1–GFP and Bik–RFP to label the microtubule extremities (Fig. 4F). Occasionally, events of vesicle movement matching microtubule plus-end dynamics could indeed be visualized (arrow), suggesting a possible role for microtubules plus-end in enhancing and/or orienting vesicular transport.

### Rho1 restores proper Snc1 trafficking and promotes the loading of Bik1 onto microtubule plus-ends

**Fig. 5.**
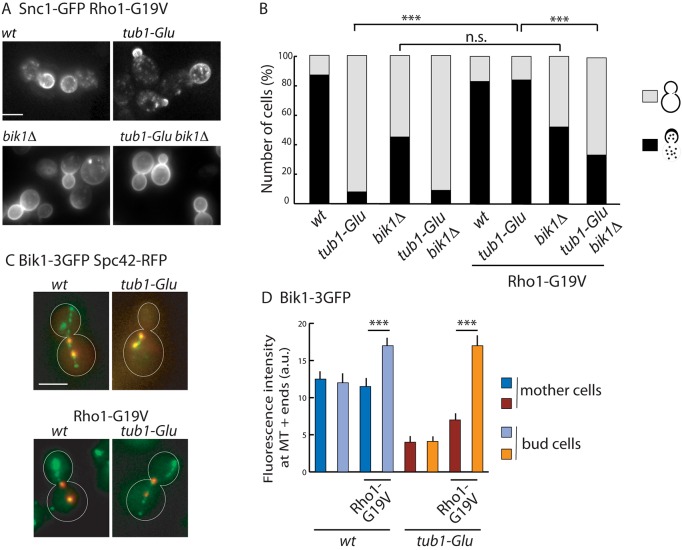
**Constitutively active Rho1 restores GFP–Snc1 transport and Bik1 association to microtubule plus-ends.** (A) Effect of Rho1-G19V expression on GFP–Snc1 localization in different strains as indicated. Rho1-G19V rescue of Snc1 misrouting is dependent on the presence of Bik1. (B) Quantification of the localization of GFP–Snc1 pattern either at the bud plasma membrane and in cytoplasmic dots, or at mother and bud plasma membranes in the different strains (*wt*, *n*=60; *tub1-Glu*, *n*=51; *bik1Δ*, *n*=59; *tub1-Glu bik1Δ*, *n*=45; *wt-Rho1-G19V*, *n*=55; *tub1-Glu-Rho1-G19V*, *n*=131; *bik1Δ- Rho1-G19V*, *n*=144; *tub1-Glu bik1Δ Rho1-G19V*, *n*=183 cells). ***P<0.0001; n.s., not significant (Fisher's exact test). (C) Distribution of Bik1–3GFP in *wt* and *tub1-Glu* cells expressing Rho1-G19V. The spindle pole body was labeled by co-expression of the Spc42–RFP protein. (D) Quantification of Bik1 fluorescence intensity at microtubule plus-ends in mother or bud cells (without and with Rho1-G19V, respectively: *wt*, *n*=49 and 69 cells, *tub1-Glu*, *n*=56 and 78 cells). Results are mean±s.e.m. ***P<0.0001 (two-tailed Mann and Whitney test). Scale bars: 5 µm.

Our screen identified synthetic growth defect between *FKS1* and the *tub1-Glu* mutation (Table S1). Fks1, together with the small GTPase Rho1, is one of the two subunits of the 1,3-β-D-glucan synthase that catalyzes the synthesis of 1,3-β-linked glucan, a major structural component of the yeast cell wall (Qadota et al., 1996). Besides this role in β-1,3-glucan production, recent data have also established a role for Fks1 and Rho1 in clathrin-dependent and/or -independent endocytosis (deHart et al., 2003; Prosser et al., 2011). This led us to test a possible implication of a Rho1-dependent mechanism in Snc1 trafficking. To test this hypothesis, Rho1 was expressed in a constitutively active form (Rho1-G19V) in the *wt* and *tub1-Glu* strains. Analysis of Snc1 localization in these two backgrounds indicated that the constitutively active Rho1 was a suppressor of the *tub1-Glu* mutation for Snc1 trafficking. Indeed, whereas the expression of Rho1-G19V in the *wt* strain had no significant effect on GFP–Snc1 distribution, its expression in *tub1-Glu* cells was sufficient to restore a normal GFP–Snc1 trafficking, with 84% of the mutant cells now harboring a *wt* phenotype (Fig. 5A,B). Interestingly, disruption of *BIK1* in the *tub1-Glu* strain strongly reduced Rho-G19V-mediated rescue of Snc1 misrouting. Along the same line, Rho1-G19V did not complement the Snc1 localization defect in the *BIK1*-deleted strain, indicating that Rho1 suppressor effect requires functional Bik1.

This observation led us to analyze the impact of Rho1-G19V expression on Bik1 localization at microtubule plus-ends using Bik1–3GFP as a reporter. In the presence of Rho1-G19V, Bik1–3GFP fluorescence at *wt* microtubule plus-ends was markedly enhanced (Fig. 5C,D). Furthermore, we found that Rho1-G19V was able to restore the localization of Bik1–3GFP to microtubule plus-ends in *tub1-Glu* strain (Fig. 5C,D). In both strains, Rho1 activation induced a preferential accumulation of Bik1–3GFP at microtubules plus-ends within the bud (Fig. 5D). Therefore, constitutively active Rho1 also functions as a suppressor of the *tub1-Glu* mutation for Bik1 localization.

Taken together, our results argue for a detrimental role for Bik1 in Snc1 trafficking, likely dependent on its localization at microtubule plus-ends the control of the GTPase Rho1.

## DISCUSSION

This report is the first comprehensive genetic analysis of a tubulin variant, used to model the accumulation of Glu-tubulin and thereby investigate the function of the C-terminal aromatic amino acid of α-tubulin. Identification of genes essential for viability or fitness of the Glu-tubulin mutant as being connected to endocytosis-associated processes led us to reconsider a possible role for microtubules in vesicular trafficking in budding yeast. Indeed, in mammals, microtubules play a well-established role in the organization of the Golgi as well as in the movement of maturing endocytic compartments, providing tracks between the cell periphery and the perinuclear region (Lowe, 2011; Thyberg and Moskalewski, 1999). In yeast, studies using pharmacological inhibitors or point-mutations affecting microtubule stability have indicated a role of microtubules in the three-dimensional configuration of the tubular Golgi network (Rambourg et al., 1996) but no major contribution to vesicular trafficking (Huffaker et al., 1988; Jacobs et al., 1988; Kubler and Riezman, 1993; Penalver et al., 1997; Rambourg et al., 1996). Our detailed analysis of the *tub1-Glu* strain supports such a role for microtubules in the Golgi organization but most importantly, it revealed defects in the localization of Abp1, with abnormal Abp1-positive comet tail structures, and of the v-SNARE protein Snc1. We established that the Snc1 trafficking defect is also obtained by deletion of the microtubule +Tip protein Bik1 and that the *tub1-Glu* phenotype can be complemented by expression of a constitutively active Rho1, which restores Bik1 recruitment at the plus-ends of microtubules. To our knowledge, these data are the first evidence of a role for the microtubule plus-ends in aspects of vesicular trafficking in *S. cerevisiae*.

Our detailed analysis of the *tub1-Glu* strain revealed a routing defect of the v-SNARE protein Snc1. This anomaly was particularly visible during budding. At this step, the protein normally accumulates at the bud membrane due to an intense exocytic activity polarized in the direction of the bud and to an efficient endocytosis and recycling back to the trans-Golgi network, preventing its diffusion from the bud to the mother cell membrane (Valdez-Taubas and Pelham, 2003). Deletion of the C-terminal aromatic residue of α-tubulin or of the protein Bik1 markedly impaired Snc1 polarized distribution at the bud. Phenotypic similarities with mutants affected in the endocytic machinery, such as *end3Δ*, suggested that the *tub1-Glu* and *bik1*Δ mutations could similarly interfere with normal uptake and trafficking of Snc1. Unexpectedly, other cargoes of the plasma membrane also internalized in the endocytic pathway but rather directed to the vacuole were not visibly affected by the *tub1-Glu* mutant (our data on Jen1) or by the use of microtubule-destabilizing conditions (drugs and temperature-sensitive mutations) (Kubler and Riezman, 1993; Penalver et al., 1997), indicating an apparent specificity of this microtubule-plus-end- and Bik1-dependent mechanism towards Snc1 or the Snc1 route. Several models, which are not necessarily mutually exclusive, could be proposed regarding the role of microtubule plus-ends in this context. A first hypothesis is that microtubules in yeast play a role as trafficking facilitators through their plus-ends, rather than as tracks per se. In this model, microtubule dynamics with continuous oscillations between growth and shrinking would generate fluxes facilitating vesicle movement. This is supported by the observation of some cases of vesicle movement following microtubule plus-ends (Fig. 4F). The protein Bik1, which has been shown to interact with a large panel of endocytic proteins (Wang et al., 2012), could provide a molecular link between microtubules and vesicles, most importantly at the microtubule plus-ends where Bik1 is enriched. Taken together, low-affinity interactions between microtubule plus-end tracking Bik1 and vesicular proteins coupled to microtubule dynamics might directly favor vesicle displacement, in a manner dependent on the overall composition of the vesicles in terms of cargoes and associated cytosolic partners and their ability to interact with Bik1. Alternatively, in the vicinity of the bud plasma membrane, where microtubule plus-ends are targeted, they could directly contribute to the assembly of signaling platforms. Snc1-specific endocytic adaptors or regulatory proteins that provide Snc1 with appropriate sorting determinants (Whitfield et al., 2016) could be part of the recruited actors, thereby favoring subsequent uptake of Snc1 (and possibly other cargoes sharing the same endocytic machinery) in the endocytic pathway. Identification of the repertoire of cargoes sensitive to the *tub1-Glu* mutation, their trafficking adaptors and the sorting motifs (possibly including post-translational modifications) responsible for their entry and routing along the endocytic pathway will be key in further understanding this new function of microtubules.

Given the functional conservation between the yeast Bik1 and mammalian CLIP170, it is reasonable to propose the existence, in mammals, of a similar CLIP170-dependent facilitating or signaling role for microtubules plus-ends that would add to microtubule tracks classical motor-dependent function in vesicle trafficking, and possibly fulfill distinct requirements in terms of trafficking distance, localization and efficiency.

Of note, the impact of the *bik1*Δ mutation on Snc1 distribution was less pronounced than that of the *tub1-Glu* mutation (Fig. 4). Even though we cannot exclude the addition of a dominant-negative effect of Bik1 due to the mislocalization of the protein in the *tub1-Glu* strain, these data might indicate that the function of the C-terminal aromatic residue of α-tubulin extends beyond the sole recruitment of Bik1. The p150^Glued^ yeast ortholog Nip100, another member of the CAP-Gly +Tip protein family, is an interesting candidate that shares properties with Bik1 and could carry out similar functions. Likewise, binding of Nip100 to microtubules might be affected by the deletion of the C-terminal aromatic residue and a number of its identified partners belong to the endocytic machinery (Wang et al., 2012). Whether deletion of *NIP100* is associated with defects in vesicular trafficking remains to be investigated.

In our report, several pieces of evidence indicate a role for Rho1-dependent signaling in Bik1-mediated microtubule functions. First, in our genetic screen with *tub1-Glu* we identified the protein Fks1, the Rho1-associated catalytic subunit of the β(1-3) glucan synthase. Second, constitutively active Rho1 allows Bik1 recruitment at the plus-end of Glu-microtubules and complements the trafficking defect of Snc1 in the *tub1-Glu* strain. Finally, Bik1 is mostly recruited on microtubules plus-ends within the bud in conditions of Rho1 activation. How Rho-GTPases achieve such regulation is currently unknown. The GTP-bound form of Rho GTPases binds a variety of partners including kinases and scaffolding proteins. As both Bik1 and CLIP170 are phosphoproteins, and as phosphorylation of CLIP170 has been shown to control its association to microtubule plus-ends (Lee et al., 2010; Nakano et al., 2010), a simple hypothesis is to propose that Rho1 controls the phosphorylation state of Bik1 through the recruitment of a specific kinase, thereby tuning its association with microtubules. In mammals, the association of the Bik1 ortholog, CLIP170, to microtubules is modulated by IQGAP1, an effector of the Rho-family GTPases Cdc42 and Rac1 (Fukata et al., 2002). Both Rho GTPases and the microtubule detyrosination and tyrosination cycle could tune the amount of CLIP170 (and possibly other +Tip CAP-Gly family members) on microtubules. The role of Rho GTPases in the recruitment of Bik1 and likely CLIP170 on microtubules, coupled to its well-established function in the organization of the actin network, could permit a joint regulation of these two cytoskeletons at specific sites, such as the bud tip or the growth cone of differentiating neurons, requiring active and efficient membrane delivery.

In addition, Rho1 has recently been shown to be a key player in endocytosis (deHart et al., 2003; Prosser et al., 2011). In our work, in its constitutively active form, Rho1 could promote a general increase in clathrin-dependent and/or independent endocytic activity, enhancing Snc1 recycling. This hypothesis would account for the partial rescue of Snc1 distribution in the *tub1-Glu bik1*Δ mutant, despite the absence of Bik1.

The list of the seven genes obtained from our synthetic lethal screen that are required for viability of the *tub1-Glu* mutant strain includes the proteins Vps15 and Cdc50. The protein kinase Vps15 is a regulator of the phosphatidylinositol 3-kinase Vps34. Phosphoinositides are key players controlling membrane trafficking dynamics through the recruitment and/or activation of unique sets of effectors. The phosphatidylinositol 3-phosphate [PI(3)P] generated upon Vps34 activation is a major determinant of endosome identity. Interestingly, Bik1 has previously been isolated as a genetic partner (synthetic lethality) of two other proteins involved in phosphoinositide synthesis, the PI(3)P 5-kinase Fab1p that converts PI(3)P into phosphatidylinositol 3,5-bisphosphate [PI(3,5)P2] on the endosomal membrane and Inp52, an inositol polyphosphate 5-phosphatase that regulates the pool of phosphatidylinositol 4,5-bisphosphate [PI(4,5)P2] (Tong et al., 2004). The rationale behind these genetic interactions is currently not clear, but one might propose that low levels of deregulation in the phosphoinositide synthesis in the context of reduced trafficking efficiency (*tub1-Glu* strain) might be sufficient to lead to cell death. The protein Cdc50 is the non-catalytic component of the Drs2 P4-ATPase that catalyzes transport of phospholipids across cellular bilayers (Lenoir et al., 2009). This flippase has been proposed to drive lipid organization and membrane deformation needed for protein recycling from the early endosome to the trans-Golgi (Furuta et al., 2007). Interestingly, Cdc50 physically interacts with the F-box-containing protein Rcy1, a partner of Snc1 (Chen et al., 2011; Hanamatsu et al., 2014). Impairment in the early endosome to trans-Golgi step in the *cdc50Δ* strain could sufficiently weaken trafficking efficiency or signaling to compromise cell viability when associated with the microtubule-driven trafficking impairment in the *tub1-Glu* strain. Analysis of Vps15 and Cdc50 and the associated signaling pathways in closer detail, might unveil unsuspected links with microtubule-driven mechanisms.

To conclude, this work clearly established a new role for microtubule plus-ends in Snc1 trafficking, and future studies will challenge the generality of such function.

## MATERIALS AND METHODS

### Yeast strains and plasmids

Strains used in this study are described in Table S2. Of note, for *wt*, *BIK1*, *BIM1* and *DYN1* deletions, two genetic background were used namely S288C (*MATα*, *ura3-52*, *lys2-801*, *ade2-101*, *trp1-Δ63*, *his1-Δ200*, *leu2-Δ1*, *tub1::HIS3-TUB1-LEU2*, *tub3::TRP1*) and BY4741 (*MATα*, *his3Δ*, *leu2Δ0*, *lys2Δ0*, *ura3Δ0*), both displaying similar localization patterns of GFP–Snc1. Snc1–GFP levels were checked to be similar in all the above strains by quantitative western blotting. The cold-sensitive β-tubulin *tub2-401* strain and microtubule stable *tub2-C354S* strains were gifts from David Botstein (Lewis-Sigler Institute for Integrative Genomics, Princeton University, NJ ) (Huffaker et al., 1988) and Mohan Gupta (Genetics Development and Cell Biology Department, Iowa State University, IA) (Gupta et al., 2002).

Cells were grown in yeast extract, peptone, glucose (YPD) rich medium, or in synthetic complete (SC) medium containing 2% (w/vol) glucose, or 0.5% (vol/vol) Na-lactate, pH 5.0 (Formedium). To address Jen1 trafficking, cells were grown overnight in SC-lactate and harvested in early exponential phase (A_600 nm_=0.3). Glucose was added to a final concentration of 2% (w/vol) and cells were maintained in these conditions for the indicated times.

The GFP–Snc1 construct was obtained from Kazuma Tanaka (Division of Molecular Interaction, Institute for Genetic Medicine, Hokkaido University, Sapporo, Japan) (Saito et al., 2004), Rho1-G19V from Yoshikazu Ohya (Department of Integrated Biosciences, University of Tokyo, Tokyo, Japan) (Sekiya-Kawasaki et al., 2002), and Jen1–GFP and Sec7–RFP from Sebastien Léon (Equipe Trafic membranaire, ubiquitine et signalisation, Institut Jacques Monod, Université Paris Diderot, Paris, France) (Becuwe et al., 2012). pLactC2-GFP was provided by Addgene. Bik1–RFP was obtained by replacement of the GFP cassette by the yemRFP cassette (Keppler-Ross et al., 2008) in pB681 (Badin-Larcon et al., 2004). For the two-hybrid experiments, the *TUB1* and *tub1-Glu* genes, from pRB539 and pRB539Glu (Badin-Larcon et al., 2004), were cloned in the pLexA vector (Addgene) in fusion with the DNA-binding domain of LexA. Bik1 and Bim1 genomic DNA were cloned into the pGADT7 vector (Invitrogen) in fusion with the GAL4 activating domain.

### Synthetic lethal screen

The *tub1-Glu* strain (ORT4557: *MATalpha-P10LEU2*, *trp1Δ63*, *leu2Δ0*, *ura3Δ851*, *arg8Δ0*, *met14Δ0*, *lys2Δ202*, *his3Δ200*, *tub3::HIS3*, *tub1-Glu::URA3*, BY4741 background) was crossed to *MATa* haploids (*MATa*, *his3Δ1*, *leu2Δ0*, *met15Δ0*, *ura3Δ0*, *GenX::Kan^R^*) from the deletion collection (Winzeler et al., 1999) in 100 µl of YPD and grown for 3 days at 30°C (Loeillet et al., 2005). The resulting diploids were then selected by transfer to 1 ml of synthetic medium (YNB, ammonium sulphate and dextrose) complemented with leucine (60 mg/l) for 4 days at 30°C, washed and transferred to 400 µl of sporulation medium (Kac 1% complemented with 60 mg/l leucine) for six days at 30°C. After sporulation, cultures were treated overnight with zymolyase 20T (ICN 0.1 mg/ml) at 30°C to kill diploids, then washed and resuspended in 500 µl of sterile water. The spores were then robotically (using a Hamilton Microlab 4000 series equipped with 12 automated needles) spotted on SC –Leu, SC –Leu +G418, SC –Leu –His +G418, SC –Leu –His –Ura +G418 plates and incubated at 30°C for 3 days. The plates were examined and compared in terms of growth phenotype. A specific lack or slow growth on the SC –Leu –His –Ura +G418 plates identifies a synthetic mutant interaction. The candidate mutants were verified upon sporulation of the double heterozygous (*gene X deleted/+*, *tub1-Glu/+*) diploids and subjected to tetrad analysis for spore germination on rich medium, observation of the size of the colonies after 3 days of growth at 30°C and genotyping of the genetic markers by replica plating on the appropriate medium with or without leucine, uracil or G418.

### Protein labeling

Jen1–GFP tagging at the endogenous locus, Jen1 trafficking and western blotting using anti-GFP (Life technology, 1:1000) were performed as described previously (Becuwe et al., 2012). For Abp1–RFP, Sla1–RFP, Sec7–RFP, Spc42–RFP and Bik1–3GFP staining, we used a direct fluorescent protein insertion at the 3′ of endogenous loci as described previously (Janke et al., 2004).

### Microscopy and image analysis

Cell imaging was performed on a Zeiss Axiovert microscope equipped with a Cool Snap ES CCD camera (Ropper Scientific). Images were captured using 2×2 binning and 12 sequential *z*-planes were collected at 0.3-µm step intervals with an exposure time of 200 ms except for time-lapse video microscopy movies of Abp1–RFP and Sla1–RFP that were collected every second with five sequential *z*-planes (0.5 µm steps) and an exposure time of 100 ms.

For analysis of microtubules and vesicle motion, cell imaging was performed on a confocal spinning disk inverted microscope (Nikon TI-E Eclipse) equipped with a Yokogawa motorized confocal head CSUX1-A1 and an Evolve EMCCD camera. A dual color acquisition of six sequential z-planes (0.3-µm steps) was performed every second with an exposure time of 50 ms and 100 ms for GFP–Snc1 and Bik1–RFP, respectively. All image manipulations, montages, and fluorescence-intensity measurements were performed using ImageJ (Schneider et al., 2012). Tracking analysis and dot number quantifications were performed using Icy (de Chaumont et al., 2012).
